# Integration of music-based game approaches with wearable devices for hand neurorehabilitation: a narrative review

**DOI:** 10.1186/s12984-024-01379-w

**Published:** 2024-05-29

**Authors:** Javier Urbina, Victoria E. Abarca, Dante A. Elias

**Affiliations:** Biomechanics and Applied Robotics Research Laboratory, Pontificia Universidad Católica del Perú, 15008 Lima, Peru

**Keywords:** Upper limb, Game, Music, Rehabilitation

## Abstract

**Background:**

Restoring hand functionality is critical for fostering independence in individuals with neurological disorders. Various therapeutic approaches have emerged to address motor function restoration, with music-based therapies demonstrating notable advantages in enhancing neuroplasticity, an integral component of neurorehabilitation. Despite the positive effects observed, there remains a gap in the literature regarding implementing music treatments in neurorehabilitation, such as Neurologic Music Therapy (NMT), especially in conjunction with emerging fields like wearable devices and game-based therapies.

**Methods:**

A literature search was conducted in various databases, including PubMed, Scopus, IEEE Xplore, and ACM Digital Library. The search was performed using a literature search methodology based on keywords. Information collected from the studies pertained to the approach used in music therapy, the design of the video games, and the types of wearable devices utilized.

**Results:**

A total of 158 articles were found, including 39 from PubMed, 34 from IEEE Xplore, 48 from Scopus, 37 from ACM Digital Library, and 35 from other sources. Duplicate entries, of which there were 41, were eliminated. In the first screening phase, 152 papers were screened for title and abstract. Subsequently, 89 articles were removed if they contained at least one exclusion criterion. Sixteen studies were considered after 63 papers had their full texts verified.

**Conclusions:**

The convergence of NMT with emerging fields, such as gamification and wearable devices designed for hand functionality, not only expands therapeutic horizons but also lays the groundwork for innovative, personalized approaches to neurorehabilitation. However, challenges persist in effectively incorporating NMT into rehabilitation programs, potentially hindering its effectiveness.

## Introduction

Neurological disorders are a group of heterogeneous diseases, some of which contribute to gait, balance, and strength problems that result in a lower quality of life. They affect nearly one billion people globally and comprise 6.3% of the worldwide disease burden [1]. Representative cases in this group are stroke, multiple sclerosis, cerebral palsy, spinal cord injury (SCI), and Parkinson’s disease (PD) [2]. These disorders can lead to significant deterioration in hand function, one of the most complex anatomical structures, and crucial in the human capacity for executing activities of daily living [3]. Particularly in individuals with these conditions, due to the significant deterioration in hand function, the ability to reach, grasp, release, and move objects effectively is frequently affected as a result of impairments of upper extremity function: reduced muscle strength, sensory loss, increased muscle stiffness, and a lack of motor control [4].

One of the primary methods of recovery after a neurological injury is neuroplasticity [5]. This phenomenon is defined as the capacity of the human brain to adapt and reconfigure its neuronal connections in response to diverse experiences [6]. When changes in the brain are connected to dysfunctional outcomes for the individual, it is termed maladaptive neural plasticity [7], such as neurological disorders. Conversely, when alterations in the brain are correlated with enhancements in an individual’s behavioral capabilities, this phenomenon is termed adaptive neural plasticity [7]. Some examples of the appearance of adaptive neural plasticity are during brain development [8] and motor skills learning [9]. As a result, ongoing research is dedicated to devising innovative treatments that can enhance neuroplasticity to rehabilitate patients with neurological disorders.

Evidence supports the effectiveness of including intensive, repetitive, challenging, and task-specific practices in interventions aimed at fostering neuroplasticity and augmenting sensorimotor recovery [10–13]. Furthermore, Petzinger et al. [12] underscore the significance of cognitive engagement as another critical component for enhancing plasticity. These authors suggest that cognitive engagement might be improved by feedback, attentional demand through cueing, and motivation. Cueing is described as utilizing external temporal or spatial stimuli to aid in initiating and sustaining movement [14].

Standard neurorehabilitation techniques for upper limb movement primarily depend on physical therapy [15]. This therapy involves targeted exercises designed to restore the functioning of the affected portion of the motor system [16]. Unfortunately, traditional occupational therapy in its current form is a process that patients dislike going through [17], as it proves difficult for them to sustain interest in repetitive exercise routines while simultaneously focusing on the precision of their movements (e.g., speed, precision, fluidity, and posture) [18, 19] and assumes greater significance considering that a patient’s attitude during physical therapy sessions is closely related to their compliance and success [20]. Children are significantly more problematic in this area since it is challenging to maintain their motivation throughout protracted therapy sessions [21]. Moreover, heightened patient motivation in their ultimate objective is a crucial facet of neuroplasticity [22]. Therefore, based on the reviewed literature, it can be inferred that effective strategies for improving neuroplasticity in neurorehabilitation should involve elements such as intensity, repetition, challenges, task-specificity, and cognitive engagement, among others.

Consequently, in upper limb neurorehabilitation, many therapy options have emerged. Lin et al. [23] realized a review of experimented training programs designed to enhance motor recovery following a stroke; these include Constraint-Induced Movement Therapy, Electromyography biofeedback, Motor imagery therapy, Robot-assisted training, Virtual reality or gaming, etc. These treatment options must incorporate the abovementioned components to promote neuroplasticity in patients.

In the quest to provide hand neurorehabilitation with intensive, repetitive, and task-specificity practices for the patient, taking into account their limitations and strengths, it is of paramount importance to assess hand functionality during therapies, as it enables the identification of changes indicating neurological decline or the tracking of responses to treatments [24]. In this regard, motion capture devices gain greater significance. Camera-based systems and keyboard-based methods, such as the Musical Instrument Digital Interface (MIDI), are widely used for movement assessment in rehabilitation. Notable studies include those employing the leap motion controller, such as a 2019 study on PD by Fernández-González et al. [25] and a study on stroke by Shah et al. [26]. Additionally, studies have used keyboards by Altenmüller in 2009 [27] and Villeneuve in 2013 [28]. However, wearable devices distinguish themselves by assessing user movement and providing assistance during rehabilitation. For instance, wearable robotic devices outperform traditional therapy due to their ability to provide a higher number of repetitions in each session, objectively assess the patient’s performance, reduce the physical strain on therapists, and enable the monitoring of the patient’s active participation in the training regimen [29]. In the same way, non-robotic wearable devices, such as data gloves, are effective instruments for tracking hand movements and evaluating hand functionality within hand rehabilitation systems [30].

One practical approach to enhancing cognitive engagement involves the use of auditory cueing. One emerging rehabilitation process that uses auditory cues is music therapy, specifically neurological music therapy (NMT) [31]. NMT is one of the few clinical interventions utilizing music as a primary rehabilitative stimulus to evoke diverse brain and motor responses [32]. Additionally, evidence suggests that musical treatments provide a therapeutic strategy for recovering functional capacities in the upper extremities of individuals with neurological disorders [33]. This is accentuated by its reliance on a research-based system of standardized clinical techniques for sensorimotor, speech/language, and cognitive training [34].

Thaut and Hoemberg [35] have classified NMT into twenty techniques, from which three address motor rehabilitation: Rhythmic Auditory Stimulation (RAS), Patterned Sensory Enhancement (PSE), and Therapeutic Instrumental Music Performance (TIMP). RAS enhances motor control by applying rhythmic sensory stimulation in rehabilitating movements with inherent biological rhythmicity, such as gait [35]. Although RAS has been extensively studied for gait neurorehabilitation, especially on PD [36], it is also applied to upper limb rehabilitation. Ghai et al. [37] realized a systematic review and meta-analysis to analyze the effects of rhythmic auditory cueing on arm function recovery post-stroke, in which beneficial effects on the Fugl Meyer test, Action reach arm test and Wolf motor time test were reported. PSE is utilized for movements that do not inherently follow a rhythm, such as typical arm and hand motions. Besides employing rhythm and timing as cues for movement, like RAS, PSE utilizes intricate patterned structures in music to organize multiple smaller motions to accomplish a more extensive sequence of movements [35]. An investigation led by Wang et al. [38] within a home-based program employing PSE reveals significant enhancements in the gross motor capacity of children with cerebral palsy. Likewise, in a recent study by Fan et al. [39], individuals with PD demonstrated enhanced speed and functionality in upper-limb movements by integrating PSE. Finally, TIMP uses musical instruments to assist patients in regaining effective movement patterns and exercising compromised motor function [35]. According to Pascual-Leone, playing a musical instrument demands extensive procedural and motor learning that results in the plastic reorganization of the human brain, which further supports the potential of music therapy in rehabilitation [40]. A systematic review performed by Yang et al. in 2022 summarizes the effect of NMT in patients with cerebral palsy. It suggests the effect of TIMP in enhancing both gross and fine motor skills, with a particular focus on improving hand function and the power associated with piano key pressing [41].

A growing aspect highlighted in Lin et al.’s [23] review of neurological rehabilitation is the incorporation of gaming, specifically the integration of gamification. Gamification is characterized as implementing game-related components in settings that extend beyond traditional gaming contexts [42]. It is suggested that incorporating gamification into neurorehabilitation has the potential to tackle various challenges related to the implementation of intensive, engaging, and cost-effective therapeutic exercises [43], even promoting motivation [44]. Games introduce challenges to patients, transforming rehabilitation into a dynamic and appealing journey [45]. Numerous research groups have advocated integrating video games as a supplementary tool alongside traditional neurorehabilitation therapy [46].

In summary, there is a need to develop innovative approaches for delivering therapeutic exercises, especially for hand neurorehabilitation. Gathering evidence in rehabilitating neurological disorders and enhancing neuroplasticity and patient adherence underscores that interventions integrating repetitive, intensive, challenging, and motivational tasks are highly likely to improve functional recovery. For this reason, this narrative review aims to investigate the effectiveness of music and game-based approaches, hand functionality assessment, and assistance by wearable devices in achieving neuroplasticity for successful hand neurorehabilitation.

## Methods

### Literature search methodology

The PRISMA [47] guidelines were followed for this literature review. This search includes refereed peer-reviewed journal papers and articles published in conference proceedings in English from 2000 to 2023, detailing the utilization of wearable devices, along with video games incorporating music therapy, to aid in rehabilitating the hand for individuals with neurological disorders. Four databases were used to conduct a literature search: PubMed, IEEE Xplore, ACM, and Scopus. The comprehensive search strategy involved a collection of primary keywords associated with “music”, “rehabilitation”, “hand”, and “game” (Table 1). Filters were applied for ACM (research articles) and Scopus (articles and conference papers).

**Table 1:** Literature search methodology

Music	music${*}$ OR music-based OR music therapy
	AND
Rehabilitation	rehabilitation OR physical therapy
	AND
Hand	hand${*}$ OR wrist OR finger${*}$ OR upper limb
	AND
Game	game${*}$ OR interface OR simulation OR virtual environment

### Eligibility criteria

The inclusion criteria for this review were meticulously tailored for journal and conference articles. For journal studies, the focus was on those that delved into neurorehabilitation, featuring interfaces that incorporated gamification approaches and were tested on defined populations, with an essential component involving music therapy. On the other hand, for conference papers, similar criteria were applied, emphasizing the incorporation of innovative gamification interfaces, empirical testing on specific populations, and integration of music therapy in the context of neurorehabilitation. The exclusion criteria, standard to journal and conference articles, pertained to studies that had not undergone peer review for publication, were published in languages other than English, took the form of books or reviews, or deviated from the primary focus on hand rehabilitation. If multiple articles by the same author or research group cover similar topics, only the most recent one will be considered.

### Studies selection

The PRISMA guidelines were used to guide the article selection process. After eliminating duplicates, the titles and abstracts of the remaining articles were reviewed. The full texts were read and selected based on the inclusion/exclusion criteria.

### Data extraction

The extracted data included author name(s) and year, the number of test subjects, participant category (with or without a neurological disorder), details on the technology used, and specific information on the type of neurological disorder (such as PD, stroke, etc.). Notably, the analysis also encompassed the extraction of insights into incorporating gamification elements and using music therapy approaches. Wearable technologies reviewed in the articles were further classified into sensor-based and robotic devices. Music therapy approaches were further analyzed to organize into RAS, PSE, or TIMP.

## Results

**Fig. 1:**
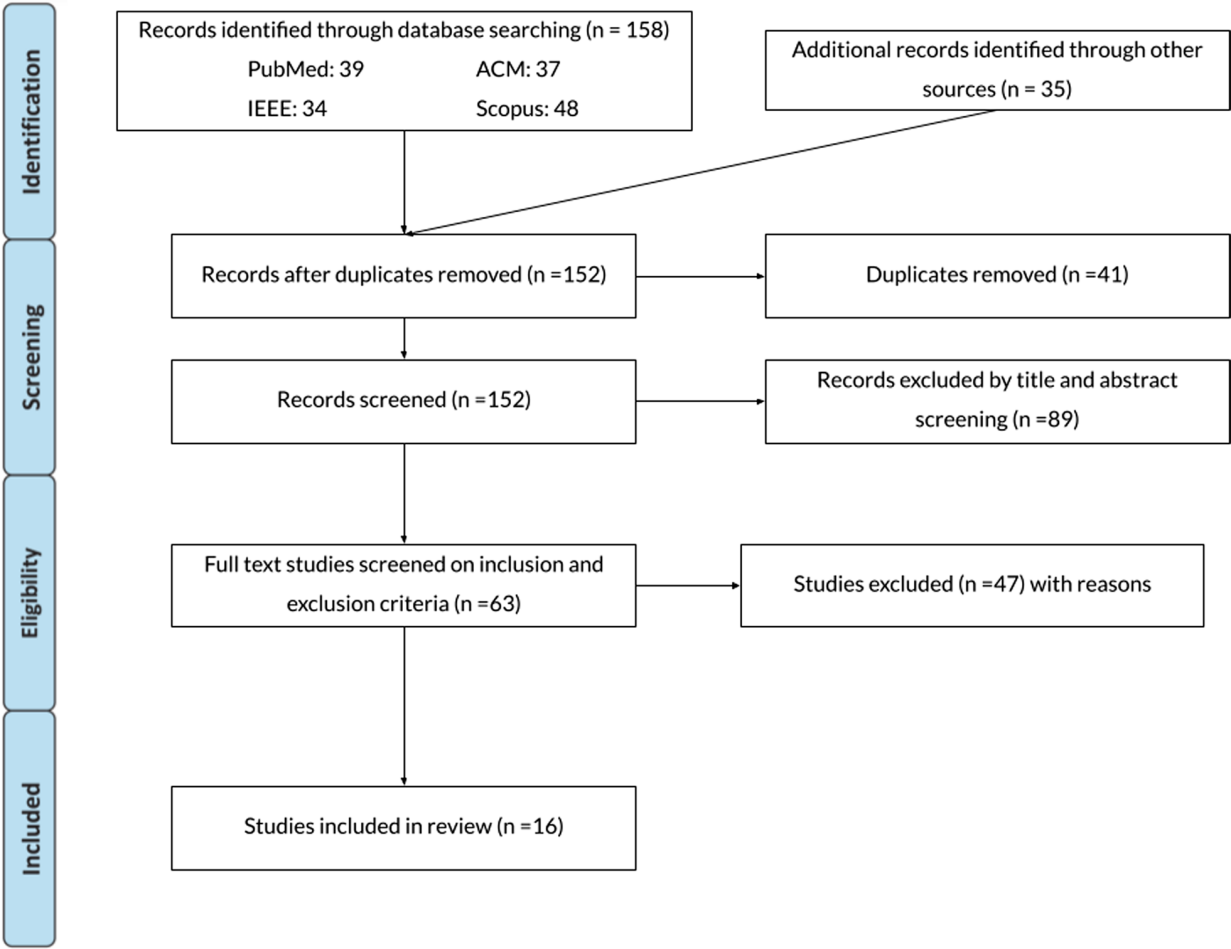
Prisma flowchart of the results from the literature search

We initially identified 158 articles through our comprehensive database search and 35 additional articles from other sources or secondary searches conducted by examining reference lists from the articles of interest and identifying studies that have cited them. After the initial screening, 41 duplicate entries were eliminated. Subsequently, we meticulously examined the titles and abstracts of the remaining 152 articles. Out of these, 89 articles were excluded due to at least one exclusion criterion. In cases where reviewers could not identify any exclusion criteria during the title and abstract screening, a thorough full-text review of the article was conducted. The total number of articles subjected to full-text assessment for eligibility was 63. Following this detailed review, 47 articles were excluded from the study. The remaining 16 articles [48–63] met inclusion criteria and were included in this systematic review (see Fig. 1 for PRISMA flowchart). The reviewed studies were eleven journals [48–51, 54, 57–60, 62, 63] and five international conferences [52, 53, 55, 56, 61]. A summary of bibliographic details and key findings from all studies is presented in Table 2. The main findings feature functional hand movement assessment tests, comprising Box and Block, 9 Hole Peg, Motor Activity Log Quality of Movement, Jebsen Test of Hand Function, and Wolf Motor Function Test. Additionally, Table 3 presents the demographic details of the studies, while Table 4 provides information on the technologies utilized.

**Table 2:** Overview of the selected studies

References	Year	Description	Main findings
Friedman et al. [53]	2011	These studies focus on the development of MusicGlove [48–51, 53]. This device targets functional movements such as pincer grip, key-pinch grip, and finger-thumb opposition while the user is playing a video game. The mechanism involves electrical leads on fingertips registering connections during specific movements. The video game consists of a modified version of the open-source game FOF. Five distinct musical notes scroll from the top to the bottom of the screen, creating the sensation of playing the song through the user’s movements. The main objective is to hit as many notes as possible. The difficulty level of each song is tailored to accommodate various degrees of hand impairment, aligning with the individual’s skill level	Effectively measures hand dexterity (% of correctly hit notes), showing a strong correlation with the BB score Incorporation of music into training sessions leads to significant improvements in both objective measures of hand motor performance and motivation
Friedman et al. [50]	2014		Participants improved hand function, particularly in grasping small objects, compared to conventional methods (BB score and 9 Hole Peg test) Strong correlations were found with the BB score Reported as more motivating than the conventional therapy
Sanders et al. [48]	2020		Amount of practice was not correlated with the average level of success experienced, but it was correlated with the amount of parameter exploration (hours of use) and completed grips were comparable to individuals in the chronic phase of stroke in a previous study [53] Feasible for autonomous home use and caused no adverse effects
Sanders et al. [49]	2022		Participants demonstrated higher compliance levels (hours of use and completed more grips) compared to individuals in previous stroke studies utilizing the same device Notable improvements in prehension ability and performance (Graded and Redefined Assessment of Strength, Sensibility, and Prehension subtests) Increased performance on the BB test compared to the conventional group
Zondervan et al. [51]	2016		Participants exhibited significantly greater enhancements in Motor Activity Log quality of movement and amount of use MusicGlove and conventional exercise groups significantly improved Box and Blocks test scores with no notable difference
Adamovich et al. [54]	2009	This study introduces a robotic/virtual environment dedicated to improving hand and arm coordination by simulating a piano with realistic visual, auditory, and tactile feedback. Enabling users to train both arms and hands concurrently, an algorithm dynamically adjusts task difficulty based on individual performance. The system supports CyberGloves for precise hand tracking and a CyberGrasp for haptic effects. The virtual piano trainer features a complete keyboard, associating each key with a corresponding sound file. Users follow configurable key sequences for songs, guided by visual cues highlighting the current key and corresponding finger. The CyberGrasp is utilized to resist flexion in inactive fingers, contributing to a comprehensive and tailored rehabilitation approach	Subjects showed improvements in both performance time and key press accuracy Two subjects improved aggregate time on the Jebsen Test of Hand Function Three out of the four subjects showed improvement in Wolf Motor Function Test aggregate time
Tingzhang et al. [56]	2014	This study comprises a sensor-based glove and computer software, creating an interactive interface with a piano for training in a home environment. The glove has five bending sensors on the fingers and integrated sensors such as a gyroscope, magnetometer, and accelerometer for precise data collection on detailed finger movements and hand position parameters that are transmitted signals via WiFi and are processed by a classifier. Correct performance triggers audio feedback, producing piano sounds corresponding to the user’s movements, and visual feedback includes an image of a marked piano with comment messages	The system is minimally intrusive, entertaining users. It operates in real time, and the classifier accurately identifies the played keys
Sun et al. [61]	2017	This study presents a wearable hand movement rehabilitation system for stroke patients, using a data glove and a keyboard game supported with hand gesture recognition. A 3D hand animation model enables patients to observe their performance during rehabilitation. The system incorporates five bend sensors, orientation sensors with 9 degrees of freedom, and gyroscopes, accelerometers, and magnetometers to capture hand movements. During the keyboard game, a metronome plays 60 beats per second, prompting subjects to alternate between different keyboard gestures every 2 s	Experimental results show high precision in recognizing simple gestures and moderate precision in complex key press gestures. The system offers user-friendly operation, avoids invasive methods, and processes data quickly
English et al. [55]	2017	This study presents an adaptive therapy gaming system designed to monitor patients’ frequency, duration, and physical motions during at-home therapy sessions. It explores the potential acceleration of motor skill learning through prior knowledge of musical cues within a robotic wrist rehabilitation system. The system utilizes an exoskeleton with a potentiometer to capture the patient’s wrist’s full range of motion, transmitting data via Bluetooth to an interactive therapy game. The game (RoboRockNRoll) encourages accurate completion of wrist therapeutic motions through strategically designed musical cues, and it involves users controlling a pick to catch music notes corresponding to popular songs during the gaming session	Participants exhibit enhanced precision in their movements when music is introduced, and this heightened precision is sustained even after the removal of musical cues. The study highlights the potential of leveraging existing knowledge of a song to facilitate users in anticipating motions, thereby accelerating the learning of a motor task Participants can acquire timing tasks more rapidly with audio-visual cues than solely on visual cues The drawback of multisensory stimuli lies in the potential impracticality of audio-visual cues in specific contexts
Xiao et al. [52]	2018	This study introduces a wrist strap incorporating FMG technology, featuring an interface to play a virtual piano. The wrist strap comprises eight FSRs and one IMU. The FSRs extract pressure patterns applied by the musculotendinous complex against the strap, while the IMU monitors arm movement with a 3-axis accelerometer, 3-axis gyroscope, and 3-axis magnetometer. The system lets users play virtual piano keys by pressing a finger on a flat surface, generating desired sounds	An initial evaluation demonstrated the practicality of utilizing FMG for virtual musical instrument control, with the user achieving successful performance within a short timeframe and encountering minimal false predictions
Taheri et al. [57]	2014	These studies present the design and initial testing of FINGER [57–59]. This device utilizes stacked single degree-of-freedom 8-bar mechanisms, individually assisting the index and middle fingers through a natural grasping motion. The mechanism’s optimization was based on trajectory data collected from healthy subjects using color-based motion capture. The rehabilitation protocol involves engaging subjects in a Guitar Hero^®^-like a game, where they play along with a song by flexing their fingers to hit notes on a visual display, receiving performance-based assistance from the robot. The study aims to test the hypothesis that optimal engagement in rehabilitation therapy occurs when subjects operate at their challenge level, achieved by controlling success rates	FINGER exhibited its capability to enable individuals with various impairment levels to engage in the game successfully FINGER, coupled with a gain-adaptation algorithm, confirmed the hypothesis that subjects could be assisted as necessary to achieve predetermined success levels in the game
Taheri et al. [58]	2012		The study observed a decrease in effort for both high and low-level subjects as their success rates increased, aligning with previous observations of user slacking when robotic assistance is excessive [57] While the study did not identify an optimal level of effort, it acknowledged the potential relationship between effort measures and the optimal challenge point, a direction for future research
Rowe et al. [59]	2017		Participants exhibited substantial improvements in functional and impairment-based motor outcomes, depression scores, and self-efficacy of hand function Notably, higher assistance levels correlated with increased motivation and secondary motor outcome enhancements, especially among individuals with more pronounced finger motor deficits
Thielbar et al. [60]	2014	Researchers developed an actuated virtual keypad (AVK) system, combining a custom actuated glove called the PneuGlove with a virtual scene consisting of a hand and five keys. This system aims to promote independence in finger movements and allows adjustments in task difficulty based on user capabilities. The PneuGlove controls air pressure to extend or prevent flexion of specific digits using air chambers, while real-time hand posture updates occur according to measured joint angles. The AVK system offers two training modes: Key Combination, which helps participants practice discrete key combinations, and Song Mode, where participants similarly play songs from the Guitar Hero video game	The AVK treatment demonstrated superiority over the intensive occupational therapy treatment for measures of ARAT and JTHFT. Additionally, changes in ARAT scores for the AVK group approached the Minimal Clinically Important Difference (MCID) of 5.7
Merians et al. [62]	2002	The study introduces a PC-based rehabilitation system that incorporates virtual reality simulation exercises. It employs the CyberGlove for free hand movement during virtual reality exercises and the RMII force feedback glove for force-exertion exercises and finger strengthening. The RMII glove, an exoskeleton device, uses non-contact sensors such as Hall-Effect and infrared to measure finger positioning and flexion. The virtual exercises are displayed on a flat screen without special 3D head-mounted displays, utilizing shadows and perspective cues for depth. The simulations involve four exercises targeting aspects of hand movement such as range, speed, fractionation, and strength. The fractionation exercise includes a piano keyboard. Before the exercises, patients’ hand movement parameters are assessed to set an initial difficulty level	The Jebsen Test of Hand Function and the Fugl-Meyer Assessment assessed each patient’s hand function improvement. Two out of three patients showed progress on the Jebsen Test, and objective measurements indicated that all patients experienced improvement in most hand parameters during the training
Mawase et al. [63]	2020	This study presents a device that measures isometric forces from each finger using a hand-shaped keyboard with ten keys, each featuring FSRs at the fingertip positions. Participants, seated comfortably facing a monitor, rest their hands on the keyboard with wrists strapped and supported by foam armrests. Only finger muscles activate the isometric forces, as forearm, arm, or trunk movements are not detectable. The device assesses finger strength and individuation through tasks that involve pressing specific keys with instructed fingers to match target force levels while maintaining low forces in non-instructed fingers	The study results indicated that finger impairment decreased following training, as assessed by the individuation task. This improvement was associated with better clinical hand function, including precision pinch performance. The training enhanced the trained task and positively impacted overall finger dexterity and movement quality, as evidenced by Fugl-Meyer and Motor Activity Log measures

*FOF* Frets on Fire, *BB* Box and Block, *FMG* force myography, *FSR* force-sensing resistors, *IMU* inertial measurement unit *FINGER* Finger Individuating Grasp Exercise Robot, *FSR* force-sensing resistors, *RMII* Rutgers Master II *ARAT* Action Research Arm Test *JTHFT* Jebsen-Taylor Hand Function Test

**Table 3:** Studies demographics

References	Year	Sample size	Sex	Age	Target population
Friedman et al. [53]	2011	10	NR	NR	Chronic stroke
Friedman et al. [50]	2014	12	F: 5 , M:7	57 ± 30.5 SD	Chronic stroke
Sanders et al. [48]	2020	11	NR	NR	Subacute stroke
Sanders et al. [49]	2022	10	F: 2 , M:8	51.3 ± 23.4 SD	SCI
Zondervan et al. [51]	2016	17	F: 7 , M:10	59.5	Chronic stroke
Adamovich et al. [54]	2009	4	NR	51.5	Chronic stroke
Tingzhang et al. [56]	2014	3 (PwoND)	NR	NR	Stroke
Sun et al. [61]	2017	10 (PwoND)	NR	NR	Stroke
English et al. [55]	2017	11 (PwoND)	F: 5 , M: 6	25 ± 5.23 SD	Upper limb impairment
Xiao et al. [52]	2018	1 (PwoND)	NR	NR	Upper limb impairment
Taheri et al. [57]	2014	16/4 (PwoND)	F: 5, M: 11/F: 1, M: 3	57.8 ± 12.5 SD/33.5 ±9.4 SD	Stroke
Taheri et al. [58]	2012	8/4 (PwoND)	F: 2, M: 6/F: 1, M: 3	56.5 ± 13.8 SD/33.5 ± 9.4 SD	Stroke
Rowe et al. [59]	2017	30	F: 10, M: 20	57 ± 13 SD	Chronic stroke
Thielbar et al. [60]	2014	14	F: 5, M: 9	57	Stroke
Merians et al. [62]	2002	3	F: 1, M: 2	65.3	Stroke
Mawase et al. [63]	2020	18	F: 5, M: 13	61.3 ± 2.1 SD	Stroke

**Table 4:** Technical data from wearable devices

References	Year	Rehabilitated target member	Wearable device	Sensor technology	System type
Friedman et al. [53]	2011	Fingers	MusicGlove (Data Glove)	Electrical leads	Passive
Friedman et al. [50]	2014				
Sanders et al. [48]	2020				
Sanders et al. [49]	2022				
Zondervan et al. [51]	2016				
Adamovich et al. [54]	2009	Fingers	CyberGlove(data glove) and CyberGrasp (exoskeleton)	Bend sensors and motion tracking	Active
Tingzhang et al. [56]	2014	Fingers	DG5-Vhand (Data Glove)	Bend sensors and IMU	Passive
Sun et al. [61]	2017	Fingers	Data Glove	Bend sensors and motion tracking	Passive
English et al. [55]	2017	Wrist	Exoskeleton	Potentiometer	Passive
Xiao et al. [52]	2018	Fingers	Instrumented strap	FSR and IMU	Passive
Taheri et al. [57]	2014	Fingers	FINGER	–	Passive and active
Taheri et al. [58]	2012				
Rowe et al. [59]	2017				
Thielbar et al. [60]	2014	Fingers	Pneuglove (pneumatically actuated glove)	Bend sensors	Active
Merians et al. [62]	2002	Fingers	CyberGlove(data glove) and Rutgers Master II-ND (exoskeleton - pneumatically actuated)	Infrared and Hall sensors	Active
Mawase et al. [63]	2020	Fingers	Hand-shaped keyboard	FSR	Passive

### Participant characteristics

The mean sample size of the included studies was 12 participants (ranging from 1 to 30 participants). Among these, ten studies exclusively involved participants with neurological disorders (PwND). Specifically SCI [49] and stroke [48, 50, 51, 53, 54, 59, 60, 62, 63]. Conversely, five studies, which were conference articles, exclusively enrolled participants without neurological disorders (PwoND). Specifically, two of these studies delved into upper limb motor impairment [52, 55] and two into stroke [56, 61]. Only two studies encompassed PwND and PwoND participants, and they centered around stroke [57, 58].

Of the eleven studies reporting participants’ ages, those involving PwND had an average age of 57, while the PwoND study reported an average age of 30. Notably, the average age of PwoND participants was lower than that of PwND participants.

### Rehabilitation environment

Most studies were conducted in outpatient rehabilitation settings for data collection, principally in laboratories [50, 53, 59] and home-based settings [48, 49, 51]. Moreover, three studies mention the device’s possible usage in home settings [55, 56, 62]. Seven studies did not state or explain the environment in which the research was conducted [52, 54, 57, 58, 60, 61, 63].

### Rehabilitation devices

**Fig. 2:**
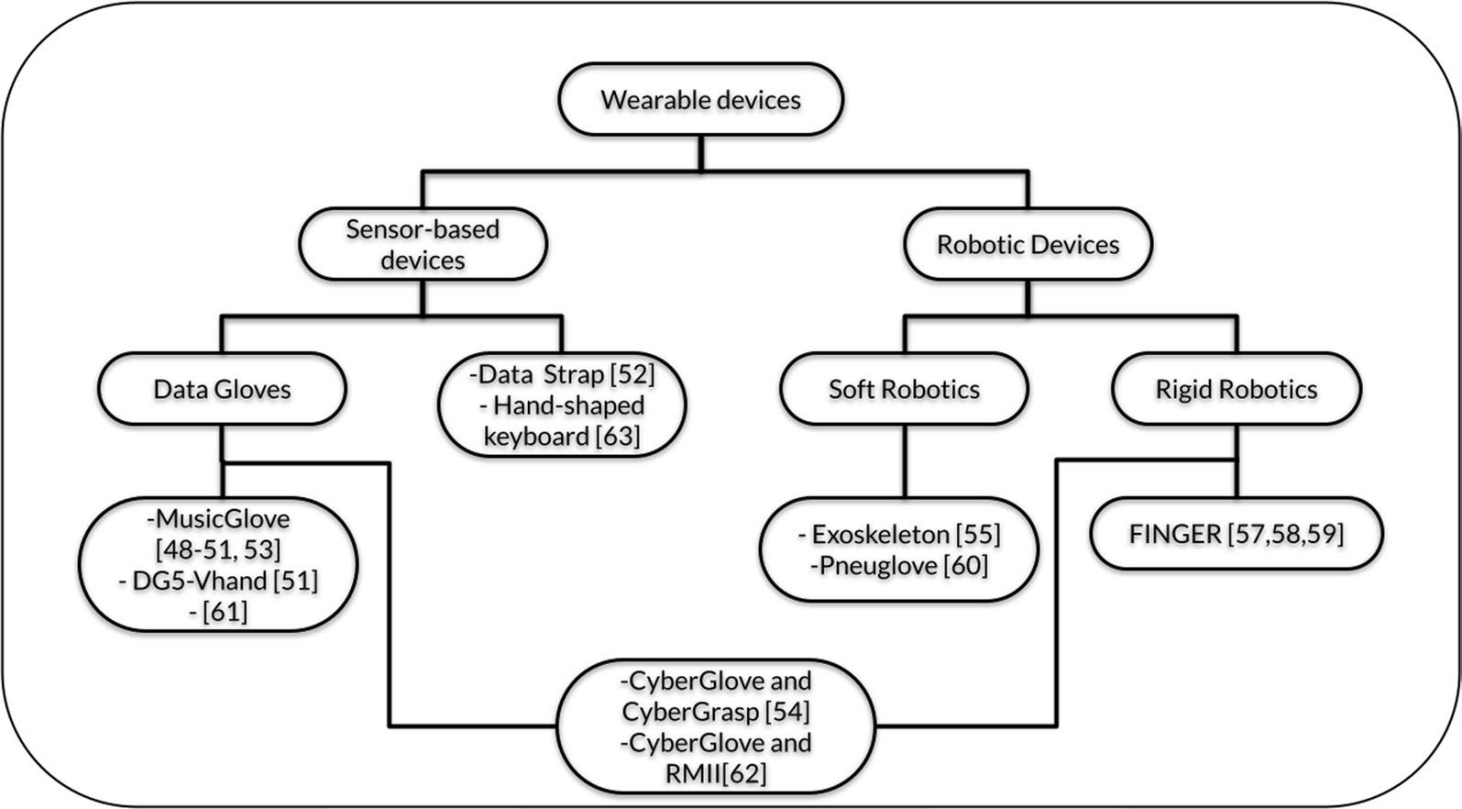
Wearable devices classification

The wearable devices in these studies have a controller role, as the user’s movements are translated into game commands that enable user control by accurately detecting and monitoring the motion of patients during exercises. Figure 2 illustrates how the wearable devices were categorized based on the findings from the studies.

Sensor-based devices utilize various sensors to monitor and collect data on a user’s motor movements or physical activities. The primary goal of such devices is to capture and analyze relevant biomechanical information, allowing healthcare professionals to assess and track the progress of individuals undergoing motor rehabilitation. Most studies employed wearable sensor-based devices: seven employed the data gloves [48–51, 53, 56, 61] and one an instrumented strap [52]. MusicGlove is the most frequently used device, and its functionality was assessed in diverse neurological disorders [48–51, 53]. Mawase et al. designed a hand-shaped keyboard that allowed participants to place their hands on the keys, with each finger gently touching a key. The setup included straps securing the wrists to a wrist rest and foam armrests supporting the forearms. This arrangement meant that the only way to generate isometric forces at the fingertips was through activating the finger muscles [63].

Wearable robotic devices are believed to enhance therapy with features like intensified sessions, feedback mechanisms, and tailored patient-specific interventions [64]. However, the rigid materials, primarily metals, used in conventional robotics to provide a rigid framework for assisting in motor function may constrain user motion. In contrast, soft robotics, crafted from deformable materials, reduce constraints on motion and joint alignment issues [64]. Their flexibility and simplicity make them lighter and more portable. As robotic devices can assess hand functionality and assist user movements, they can be categorized as active or passive. An active system necessarily includes an actuator and aids the user in performing specific movements. In contrast, passive robotic devices do not actively assist or resist user movements; instead, they are often used to support or stabilize a body part during rehabilitation exercises. Given this definition, sensor-based devices can be classified as passive since they primarily monitor user movement and can offer stability through static supports, though less intricate than exoskeletons. For instance, the device developed by English et al. [55] is a passive robotic system that employs a soft exoskeleton to guide the user in focusing solely on wrist movement. While the arm remains stable, the device supports and stabilizes the user during wrist exercises. On the other hand, the FINGER (Finger Individuating Grasp Exercise Robot) device is primarily active, functioning as a rigid exoskeleton. Still, its dual assistive function will be discussed later.

In the literature search, two studies employed soft robotics devices: one employed an exoskeleton [55], and one used a pneumatically actuated glove, an active device. It can be used to extend (or prevent flexion) a specified digit by inflating an air chamber located on the palmar side of the digit [60]. Four studies employed rigid robotic devices: the FINGER robotic exoskeleton [57–59], and the RMII (Rutgers Master II) pneumatically actuated exoskeleton [62]. The devices developed by Adamovich et al. [54] and Merians et al. [62] were the only studies that employed a data glove (CyberGlove) for hand tracking, as well as an exoskeleton (CyberGrasp [54] and RMII [62]) for haptic effects. Despite not initiating user movement on their own, these devices are classified as active because they assist the user by restricting finger flexion, except for the targeted finger, thereby facilitating rehabilitation.

### Sensing technologies

Three categories could be used to group the involved sensing techniques: motion tracking sensors (gyroscope, magnetometer, accelerometer, infrared and Hall sensors or inertial measurement unit (IMU)) [52, 54, 56, 61, 62], flexible sensors [54, 56, 60, 61] and others. The first category measures complicated body posture, joint range of motion, and kinematic characteristics, including orientation, location, and velocity [65]. Regarding the second category, this sensor’s degree or radius of deformation results in a proportional resistance output. The last category includes: electrical leads [48–51, 53], force sensing resistors (FSR) [52, 63], and a potentiometer [55]. Concerning the rigid robotic devices, the FINGER device [57–59] incorporates an accelerometer, not for assessing user hand movement, but as an integral component of the robot control system. This device indirectly measures the force the subjects apply, capturing the force exerted against the robot. Furthermore, eight studies employed more than one sensing technology [52, 54, 56–59, 61, 62].

### Gamification approaches

Between the studies, the most commonly used video games are similar to Guitar Hero^®^. This popular rhythm-based video game lets players simulate guitar playing using a specialized guitar-shaped controller. Through a scrolling interface showing colored notes matching different frets on a virtual guitar neck, players must press corresponding buttons and strum in sync with the flowing notes, mirroring the rhythm of songs. The aim is to accurately hit notes to earn points and progress through songs, with advancing levels offering more challenging tracks that test players’ coordination, timing, and musical skills. Video games developed based on Guitar Hero include Frets on Fire (FOF) [48–51, 53], RoboRockNRoll [55], and games used by the FINGER robot [57–59] and by Thielbar et al. [60]. These games were adapted to users’ capabilities and the target rehabilitation member. The RoboRockNRoll video game is the only one that aims to encourage wrist movement by encouraging flexion and extension.

Another commonly employed method involved the use of virtual pianos. Xiao et al. [52], Zhang et al. [56], and Mawase et al. [63] designed virtual pianos with comparable attributes, providing visual and auditory feedback by incorporating markers on the pressed keys and corresponding musical cues. In contrast to the previously presented video game-based studies, Adamovich et al. [54], Thielbar et al. [60], Merians et al. [62], and Sun et al. [61] stand out as the only studies adopting a virtual reality approach. Adamovich et al. crafted a piano simulation featuring virtual hands from a first-person perspective, asserting its superior intuitiveness compared to the third-person perspective [54]. It integrates visual and auditory feedback, such as studies employing a virtual piano. However, it uniquely incorporates haptic feedback facilitated by its exoskeleton.

## Discussion

This paper reviews the featured technologies developed over the recent years, focusing on music-based games using wearable devices for rehabilitation. The studies included in this review demonstrate differences in terms of the age of participants, target population, sample size, and sensor technologies employed. Given the lack of studies that complemented wearable devices with music-based games for motor rehabilitation therapies, such diversity in participant characteristics was anticipated.

### Home-based rehabilitation and wearable devices

The literature review reveals that a substantial portion of these investigations have been executed with the aim of outpatient rehabilitation settings. Their explicit focus on developing and assessing rehabilitation devices to cater to home-based usage sets many of these studies apart. This shift towards a home-based context augments user engagement and commitment to the therapeutic process and transcends the spatial limitations imposed by traditional clinic-centric rehabilitation methodologies [66], thereby enhancing long-term adherence and fostering a sense of autonomy in the rehabilitation journey.

Furthermore, it is noteworthy that the rehabilitation device predominantly featured in these studies is the MusicGlove. This inclination towards MusicGlove finds resonance in its precedent evaluation within controlled laboratory settings, as documented in works such as [50, 53]. Notably, these lab-based evaluations also encompassed comprehensive assessment reports, a pivotal element that contributes to user acceptance and guides the development of the device. This meticulous approach to evaluation has carried over to the home-based studies, as exemplified by references such as [48, 49, 51], wherein no adverse effects have been reported.

### Rehabilitation approaches performed by wearable devices

Sensor-based devices used in this review are produced to be compact and light, enabling users to wear them in various settings without being intrusive and allowing them to interact with the video game appropriately. As previously noted, these devices employ a passive rehabilitation approach that does not include actuators but relies solely on sensing technologies. This category includes data gloves [48–51, 53, 56, 61] and data straps [52], which don’t offer movement assistance. In other words, these devices are designed to assess user performance through sensor technologies and gauge the impact of the associated video game’s cues, feedback, and motivation. A noteworthy study of sensor-based devices is the hand-shaped keyboard developed by Mawase et al. [63]. This keyboard offers improved ergonomics by supporting the wrist and forearm, allowing users to channel force exertion more efficiently into their fingers.

The only studies that solely use a rigid robotic device are those by [57–59], which utilize the FINGER device. This active device aids the user by providing resistance against the flexion of non-targeted fingers. This device’s dual rehabilitation approach combines passive and active elements. It begins with the user independently performing finger flexion, a passive aspect of the process. Once the user reaches a specific flexion angle threshold, the active component is introduced, as the device provides performance-based assistance from the robot.

Soft robotic devices don’t employ traditional rigid actuators like hydraulic and pneumatic. Instead, they integrate lightweight actuators sensing technologies for movement assistance and sensor technology for movement assessment. The exoskeleton developed by [55] serves a crucial support function, specifically aiding users in executing wrist movements while maintaining stability. The study by Thielbar et al. [60] uses the Pneuglove, a pneumatic exoskeleton, and the CyberGlove, which was solely utilized for outcome measurement and not as a video game interface. The Pneuglove is an active device that prevents the flexion of a specific finger and initiates the user’s finger flexion movement independently. These two modes correspond to the video game’s two gameplay modes: the first mode prevents finger flexion for the keyboard game, while the second mode, based on Guitar Hero, assists the user in flexing their finger to strike musical notes. These functionalities enable the device to support a broader range of movements, aiding users across different game modes.

Two studies [54, 62] used a combination of sensor-based and robotic devices, offering an active rehabilitation approach by applying pressure on the targeted finger during key movements. Though they utilized both devices, they always worked together, unlike the FINGER device, which combines both active and passive approaches. The force exerted by the actuators can be finely adjusted, even down to a minimal level. In such cases, the assistance can be considered primarily passive, aligning with an advanced stage of rehabilitation where the user requires less aid.

Most of the devices focus on finger rehabilitation, with only one dedicated to wrist rehabilitation [55], and were tested exclusively on PwoND. Moreover, no device addressed the rehabilitation needs of both the fingers and the wrist. This is notable because various conditions, like Carpal Tunnel Syndrome-a prevalent neurological disorder-often involve simultaneous issues in both the fingers and the wrist. In cases like Carpal Tunnel Syndrome, pressure or constriction at the wrist affects the median nerve, extending from the forearm to the palm [67].

### Motion tracking technologies for enhanced rehabilitation monitoring

In various studies, sensor technologies’ primary focus is precisely detecting in real-time user performance monitoring during movements. However, a case in point is the MusicGlove system, which, due to its reliance on contact sensing pads, imposes restrictions on the required movements for completion. This constraint results in a fixed set of ”acceptable movements,” limiting the system’s adaptability. MusicGlove provides feedback solely on movement completion, lacking real-time position data.

Various electronic sensors and systems have been employed in these studies, including accelerometers, gyroscopes, magnetometers, IMUs, FSRs, electrical leads, and potentiometers. Motion tracking sensors such as accelerometers, gyroscopes, magnetometers, and IMUs are essential in virtual reality video game studies. These sensors enable the development of 3D hand animation models and support hand motions across multiple axes, which is necessary for virtual piano applications. This approach helps create a sense of immersion and realism for the user, as recommended by [54].

### Musical themes and adaptive video games

Many gamification approaches designed for a playful rehabilitation experience center around a musical theme, often involving playing the piano or catching musical notes. Most studies presented video games similar to Guitar Hero^®^, one of the most famous music video games. The primary benefits of employing commercial games lie in their widespread acceptance and budget-friendly costs. However, these games may need more comprehensive guidance or measurement features for monitoring the precise movement and positioning of the arms, hands, and fingers, limiting their effectiveness for therapeutic purposes. Therefore, the studies that employed similar versions of commercial video games realized modifications to the game, enabling it to adapt to user needs. In the case of selecting the piano in certain studies, it is suggested that this choice was made due to its familiarity among the general public.

It is crucial to allow the modification of video game parameters to achieve specific rehabilitation objectives. For example, Thielbar et al. [60] noted that the challenge of a task can be adjusted to match the user’s abilities in several ways, such as changing the level of assistance provided by the PneuGlove, altering the speed of key presses, and selecting specific key combinations for practice. This flexibility is essential when designing video games for long-term rehabilitation and self-administered care in home settings, especially in telemedicine or telerehabilitation scenarios, as mentioned by Merians et al. [62]. It is essential to balance providing an optimal challenge level and avoiding overly complex or too easy tasks, as suggested by Mihaly Csikszentmihalyi [68]. Merians et al. [62] quantified each patient’s thumb and finger range of motion, speed, fractionation, and strength before initiating exercises to establish an initial difficulty level. Since that study in 2002, more automated approaches have emerged, especially with the rapid growth of artificial intelligence applications in healthcare. Some studies have incorporated algorithms to automate the selection of challenge levels [54, 57–59].

The reviewed video games are designed to motivate users and guide the creation of either passive devices, where users independently initiate and complete movements without assistance, or active devices, which provide resistance to help guide the movement of specific body segments. The absence of devices that assist the user in initiating movement may limit the range of potential applications, as targeting neuroplasticity early in the progression of a neurological condition is crucial. Patients often require more assistance in such cases, and active devices that initiate movement can provide significant benefits.

### Music therapy insights

The consistent use of TIMP across the reviewed studies indicates a preference for a method familiar to therapists and patients, as playing musical instruments is an everyday leisure activity for many. However, TIMP differs from traditional leisure activities in that it involves playing instruments in unconventional ways. Studies focusing solely on TIMP incorporated virtual piano or keyboard elements [52, 54, 56, 62, 63]. TIMP has been linked to improvements in grip strength, finger strength, and gross and fine hand motor skills [69], as demonstrated by clinical outcomes such as the Fugl-Meyer test and Wolf Motor Function test. These functional gains underscore the efficacy of TIMP in rehabilitative settings.

Additionally, some studies incorporated RAS, which uses auditory rhythmic cues such as repetitive pulses or metrically accentuated music [70]. In video games similar to Guitar Hero, metrically accentuated music leverages specific songs and increases the tempo to heighten the challenge, encouraging users to adapt to the quicker pace. Sun et al. [61] is the only study that combined RAS and TIMP. In this study, a metronome (repetitive pulses) served as a cue to guide users in performing specific gestures, aiding in the timing of piano key pressing.

However, it’s noteworthy that only a few studies evaluate the impact of music therapy with and without incorporating video games. This evaluation extends to understanding the effects of both visual and auditory cues on users. Considering the potential impact of music therapy, it becomes crucial for a more comprehensive assessment in future studies. It would be particularly interesting for devices that offer movement assistance, such as rigid robotics, to incorporate evaluations of the impact of music therapy. Quantifying the influence of visual and auditory cues in conjunction with movement assistance could provide valuable insights into optimizing rehabilitation outcomes. The study by English et al. strongly emphasized the impact of auditory cues, examining user performance with both visual and auditory cues compared to using visual cues alone. The study concluded that combining audiovisual cues enables participants to learn timing tasks more efficiently than relying solely on visual cues. The authors also highlight the importance of choosing the appropriate cues based on the specific skills taught and the performance metrics used to measure these skills.

PSE sessions are designed to engage patients dynamically and interactively. In a PSE scenario, patients might be prompted to perform specific hand and arm movements in response to a rhythmic auditory cue, such as a steady drumbeat or a melodic sequence. This auditory guidance is a precise and structured framework, enhancing the synchronization and coordination of the patient’s movements. The rhythmic precision of the auditory cues not only aids in motor skill development but also introduces an element of enjoyment and engagement, transforming the rehabilitation process into a more immersive and stimulating experience [35].

Despite its potential benefits, the adoption of PSE in rehabilitation processes is currently limited, primarily due to the intricate nature of the approach. The practical implementation of PSE requires the expertise of a skilled Music Therapist [34] who can tailor the exercises to each patient’s individual needs and capabilities. The scarcity of PSE in current rehabilitation practices underscores the importance of specialized practitioners in unlocking the full potential of this approach. As the field progresses, further research and exploration into applying PSE in diverse rehabilitation contexts could reveal novel treatment strategies for enhancing the effectiveness of hand therapeutic interventions.

### Limitations

The search strategy was meticulously designed to encompass specific terms such as game, interface, simulation, and virtual environment. Rather than opting for the more general term ”video game,” we chose a more refined approach to capture exclusively articles that extensively discuss video games, filtering out other types of gamification methods. Although the narrative approach aligns well with the diverse descriptions of game design in the articles, it leans towards interpretation rather than strictly adhering to systematic or scoping review methodologies. The assessment of each wearable device’s effectiveness in tandem with music-based gamification approaches required nuanced interpretation, as only overarching findings were provided. Therefore, readers should look over the review’s findings with an awareness of its interpretation. Nevertheless, the narrative approach offers flexibility in contextualizing the unique features of wearable devices and the analysis of music therapy.

## Conclusion

Our review provides an overview of the transformative effects of NMT within neurorehabilitation. We have explored its synergies with emerging fields, including the gamification of physical therapies and the integration of wearable devices tailored for hand functionality and assistance. NMT appears as a promising alternative to traditional treatments, such as pharmaceutical interventions, with significant potential. Our investigation underscores the ongoing exploration of neuroplasticity enhancement in motor learning, positioning NMT as a dynamic and impactful avenue. The fusion of NMT with cutting-edge technologies expands the horizons of therapeutic interventions and lays the foundation for innovative, personalized approaches to neurorehabilitation. Nevertheless, challenges persist in integrating NMT into video games within rehabilitation programs. While many programs incorporate NMT, they often limit its application to auditory feedback alone, neglecting the full spectrum of elements that music can offer. Such simplistic implementations are less likely to elicit the desired neurobiological responses associated with NMT, potentially hindering its effectiveness. This limitation contrasts with observations in other fields, highlighting the need for a more comprehensive utilization of NMT within the context of video games for enhanced neurorehabilitation outcomes.

## Data Availability

Not applicable.
